# Metal and Metal Oxide Nanoparticle Incorporation in Polyurethane Foams: A Solution for Future Antimicrobial Materials?

**DOI:** 10.3390/polym15234570

**Published:** 2023-11-29

**Authors:** Radu Claudiu Fierascu, Eduard-Marius Lungulescu, Irina Fierascu, Miruna S. Stan, Ionela C. Voinea, Silviu Ionel Dumitrescu

**Affiliations:** 1 National Institute for Research & Development in Chemistry and Petrochemistry ICECHIM, 060021 Bucharest, Romania; fierascu.radu@icechim.ro (R.C.F.); irina.fierascu@icechim.ro (I.F.); 2 National Institute for Research and Development in Electrical Engineering ICPE-CA, 313 Splaiul Unirii, 030138 Bucharest, Romania; 3 Faculty of Horticulture, University of Agronomic Sciences and Veterinary Medicine of Bucharest, 59 Marasti Bvd., 011464 Bucharest, Romania; 4 Department of Biochemistry and Molecular Biology, Faculty of Biology, University of Bucharest, 91-95 Splaiul Independentei, 050095 Bucharest, Romania; miruna.stan@bio.unibuc.ro (M.S.S.); ionela-cristina.voinea@bio.unibuc.ro (I.C.V.); 5 Central Emergency University Military Hospital, 013058 Bucharest, Romania; dr.silviu.dumitrescu@gmail.com; 6 Medical-Surgical Department, Faculty of Medicine, Titu Maiorescu University of Medicine and Pharmacy, 031593 Bucharest, Romania

**Keywords:** polyurethane foams, metallic nanoparticles, antimicrobial properties, biomedical applications

## Abstract

With the technological developments witnessed in recent decades, nanotechnology and nanomaterials have found uses in several common applications and products we encounter daily. On the other hand, polyurethane (PU) foams represent an extremely versatile material, being widely recognized for their extensive application possibilities and possessing a multitude of fundamental attributes that enhance their broad usability across various application fields. By combining the versatility of PU with the antimicrobial properties of nanoparticles, this emerging field holds promise for addressing the urgent need for effective antimicrobial materials in various applications. In this comprehensive review, we explore the synthesis methods, properties and applications of these nanocomposite materials, shedding light on their potential role in safeguarding public health and environmental sustainability. The main focus is on PU foams containing metal and metal oxide nanoparticles, but a brief presentation of the progress documented in the last few years regarding other antimicrobial nanomaterials incorporated into such foams is also given within this review in order to obtain a larger image of the possibilities to develop improved PU foams.

## 1. Introduction

In addition to the common applications of polyurethane foams, such as insulation for walls and roofs, coatings, elastomers, adhesives and sound insulation materials in the construction and automotive sectors, efforts have been directed towards utilizing these materials in various biomedical applications, like stents, vascular prostheses, breast implants, dressings, antibacterial surfaces, catheters, controlled drug release for cancer treatment, tissue engineering, nerve regeneration, etc. [1].

Polyurethanes constitute a class of polymeric materials primarily synthesized through a polyaddition reaction involving a diisocyanate (such as methylene diphenyl diisocyanate (MDI) or toluene diisocyanate (TDI)) and a mixture of polyols (either polyether or polyester diols), conducted with the aid of catalysts (typically tertiary amines), chain extenders and blowing agents (including CO_2_ generated from the reaction of water with an NCO group, as well as pentane, among others). The final characteristics of the resulting polyurethane foam (density, mechanical strength, gelation time, pore structure) can be finely tuned by manipulating factors such as the molecular weight of the polyols, hydroxyl (OH) and isocyanate (NCO) indices, the quantity of the blowing agent, the type and amount of catalyst, the surfactant and so on [2,3].

Obtaining antimicrobial polyurethane foam involves incorporating antimicrobial agents or additives into the polyurethane foam during the manufacturing process. These agents can include metal nanoparticles, quaternary ammonium compounds or other substances known for their ability to inhibit the growth of microorganisms [4,5]. Antimicrobial additives are typically mixed with the polyurethane polymer before it is foamed and cured. This ensures that the resulting foam possesses antimicrobial properties, making it suitable for various applications, including medical devices, upholstery and filtration systems, where preventing the growth of harmful microorganisms is essential for safety and hygiene [6,7].

When it comes to the creation of polyurethane foams modified with nanometal particles, the existing literature is relatively limited. This scarcity can be attributed to the use of conventional methods for producing metal nanoparticles, which rely on aqueous solutions. It is widely acknowledged that the precise control of water is crucial in the process of synthesizing polyurethanes with the specific properties for their intended applications. Additionally, achieving an adequate dispersion of these nanoparticle systems presents certain challenges. In specific cases, PUF nanocomposite materials have been generated through different methods, including immersing PU in solutions containing metal ion precursors followed by a chemical reduction [8], immersing PU in solutions with dispersed nanoparticles [9], in situ polymerization [10] or electrospinning [11].

Conversely, an equally significant factor in shaping the characteristics of nanocomposites comprising polyurethane foam and metal nanoparticles is the management of their particle dimensions, arrangement and morphology, alongside the method of their incorporation into the polyurethane foam matrix [12,13,14].

The growing concern over antimicrobial resistance has fueled intense research into novel materials capable of combatting infectious agents. This review article delves into an intriguing prospect for the future: the incorporation of metal and metal oxide nanoparticles into polyurethane foams. By combining the versatility of polyurethane with the antimicrobial properties of nanoparticles, this emerging field holds promise for addressing the urgent need for effective antimicrobial materials in various applications. In this comprehensive review, we explore the synthesis methods, properties and applications of these nanocomposite materials, shedding light on their potential role in safeguarding public health and in environmental sustainability.

## 2. PU Foams—A Versatile and Widely Encountered Material

Polyurethane (PU) foams, widely recognized for their extensive application possibilities, possess a multitude of fundamental attributes that enhance their broad usability across various application fields.

**Figure 1:**
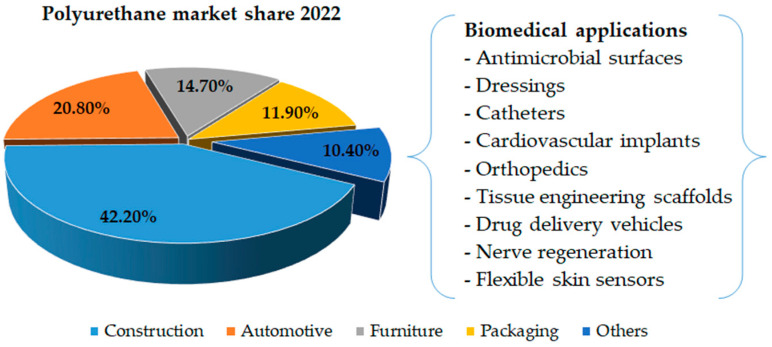
Polyurethane foam market share in 2022 and its biomedical applications (source of raw data: [15]).

As outlined in the Polyurethane Foam Market report [15], the worldwide polyurethane foam market had a valuation of USD 44.02 billion in 2022, with a forecasted trajectory of ascending to USD 64.44 billion by 2028, manifesting a compound annual growth rate (CAGR) of 5.8% within the forecast period spanning 2023 to 2028. The pronounced growth in this market is predominantly driven by the increasing demand for polyurethane foam in various industries, including the construction, automotive, furniture and packaging industries. Figure 1 provides a comprehensive visual representation of the primary applications of polyurethane foams, delineating their respective market shares within various sectors [15,16,17]. This graphical depiction highlights the distribution of polyurethane foam usage across sectors such as construction, automotive, furniture and packaging and also summarizes its application in biomedical contexts.

A notable feature of PU foams is their exceptional versatility [5,18]. They can be tailored through different formulations to acquire specific properties, be that the flexibility needed for cushioning or the stiffness essential for insulations. This inherent adaptability enables PU foams to serve in a diverse range of applications, spanning from lightweight, thermally insulating construction materials to the comforting support of mattresses [19,20,21].

This versatility allows for the incorporation of diverse fillers, a practice that extends the foams’ utility by enhancing their intrinsic properties. Among the arsenal of modifiers, flame retardants stand as a vital component, imparting increased fire resistance to these foams [22,23,24]. Different synthetic fibers (e.g., polymers, glass fibers) or natural fillers (e.g., cellulose, chitin, hazelnut and eggshell), on the other hand, reinforce the polyurethane foam’s structural integrity [25]. Incorporating antimicrobial agents into polyurethane foams provides them with the ability to combat microbial growth, a feature highly coveted in the medical and hygiene industries [26,27,28]. Furthermore, the infusion of metal nanoparticles and inorganic oxides (including nano forms) bestows advanced functionalities, e.g., enhanced thermal conductivities, superior electrical properties, improved mechanical strengths, reductions in noise pollution, and broad-spectrum antimicrobial activities [20,21,22,29,30,31].

In addition to their versatility, PU foams are well known for their lightweight nature. Their exceptional thermal insulating properties are highly regarded in the construction and refrigeration industries, where energy efficiency is a key concern [32]. The foams’ ability to dampen sound and reduce noise further broadens their appeal, with applications in automotive interiors and architectural acoustics [29,30,33].

Durability is another key facet of PU foams. They are designed to withstand wear and tear, ensuring long-lasting performance. Additionally, some formulations can exhibit resistance to various chemicals [34,35].

As sustainability and environmental concerns become increasingly important, efforts have been made to develop more eco-friendly alternatives within the area of PU foams [35,36]. This ongoing evolution seeks to reduce the environmental footprint of PU foam production and application.

## 3. Methodology

For the selection of the published works to be included in the review, the Preferred Reporting Items for Systematic Reviews and Meta-Analyses 2020 (PRISMA) recommendations were followed [37]. The research strategy was formulated according to the PICO (Problem, Intervention, Comparison, Outcome) approach (Table 1).

The research was conducted based on the PICO question: “Can metallic and metal oxide nanoparticles provide appropriate antimicrobial properties to PU foams?” As such, the following inclusion/exclusion criteria were defined.

### 3.1. Inclusion Criteria:

- Research articles published in the time interval 2012–present, full text;
- Articles published or available in English;
- Incorporation of nanomaterials—for automatic screening, only the term “nano*” was used;
- Development of materials with antimicrobial properties—for automatic screening, the automatic search “antimicrob* OR antibact* OR antifung*” was used;
- Relevance of the review topic (new information provided).

### 3.2. Exclusion Criteria:

- Articles published before 2012;
- Book chapters or books;
- Review or systematic review articles;
- Conference papers, notes, letters, short surveys, errata, editorial or conference reviews;
- Retracted papers;
- Articles published in languages other than English;
- Articles not presenting the incorporation of metal/metal oxides nanoparticles.

The literature search was conducted using the SCOPUS (as a more exhaustive database) database, using “polyurethane foams” as the primary search term. Further selection of the articles was performed automatically using the inclusion/exclusion criteria defined above, while inclusion in the present review was decided after a full review of the manuscripts.

## 4. Results

**Figure 2:**
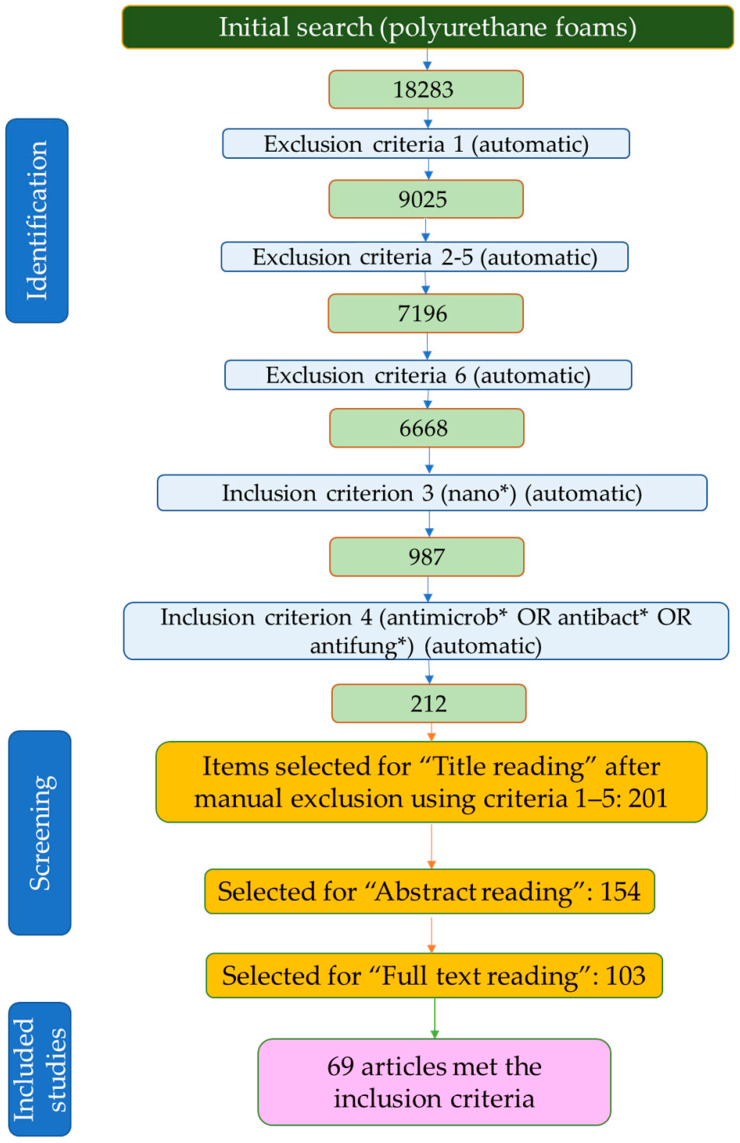
Article selection process flowchart.

After applying the above-stated exclusion and inclusion criteria, as well as reading the title, abstract, and full text, a total of 69 articles were selected for inclusion in the present review (Figure 2), covering the modification of polyurethane foams with metallic and metalloid nanoparticles. To the selected articles, other works were added to provide the necessary context. These articles were retrieved by a “search and find”/manual selection approach using the SCOPUS database (by searching using specific keywords) or were suggested by reviewers during the peer review process.

## 5. Incorporation of Metal-Based Nanomaterials into PU Foams

When speaking of antimicrobial foams, as the main focus of the present review, one of the main materials that comes to mind is silver. A well-known and widely used antimicrobial material, silver (in very different formulations) represents the subject of a multitude of patents [38] or products already on the market [39]. It is no wonder that most of the modifications presented (Table 2—presenting the incorporation of ex situ-formed nanoparticles; Table 3—presenting the in situ formation of nanoparticles in a PU matrix) are based on silver in different forms. Other known antimicrobial metals (such as copper or zinc) are also represented by a significant number of papers. However, several other types of PU foam modifications were also encountered, with, e.g., reinforcement or increasing PU foams’ fire resistance or their application in environmental protection, as their main goals. The reason for their inclusion in the present review is the intrinsic antimicrobial properties of the used nanoparticles.

According to the literature data surveyed, three main methods to develop PU foams containing different types of nanoparticles can be identified: mixing the metal salts or nanoparticle solutions in a polyol precursor, the in situ formation of nanoparticles in already constructed PU foams and, finally, physical deposition (i.e., by dipping, spraying, etc.) of NPs on PU foams. All methods have their advantages and shortcomings, which, in our opinion, should be carefully considered when selecting the composite synthesis route, together with the envisaged application. For example, physical deposition of NPs, as an advantage, preserves their morphological and physical properties, although its main disadvantage is represented by insufficient depth penetration into PU foams. The presence of the NPs mostly on the surface also represents a disadvantage of in situ formation, together with supplementary variables that influence the NP’s size and shape. The in situ formation of NPs by mixing in a polyol structure usually has, as a main advantage, a homogenous distribution in PU foams, although this, on the other hand, could affect both the properties of the PU foam and the developed NPs.

**Figure 3:**
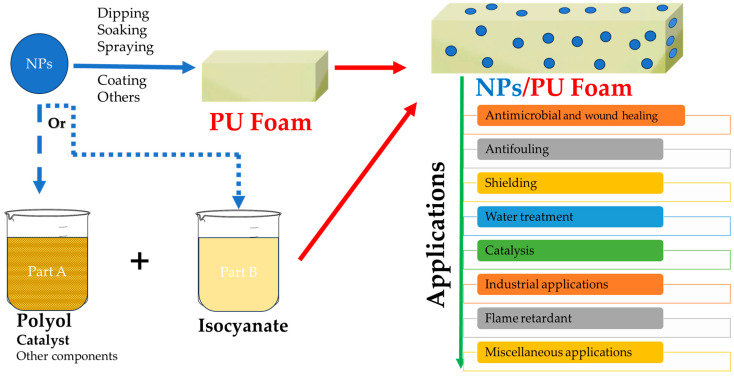
Schematic representation of NP/PU foam composite development using ex situ-synthesized NPs and its applications.

**Figure 4:**
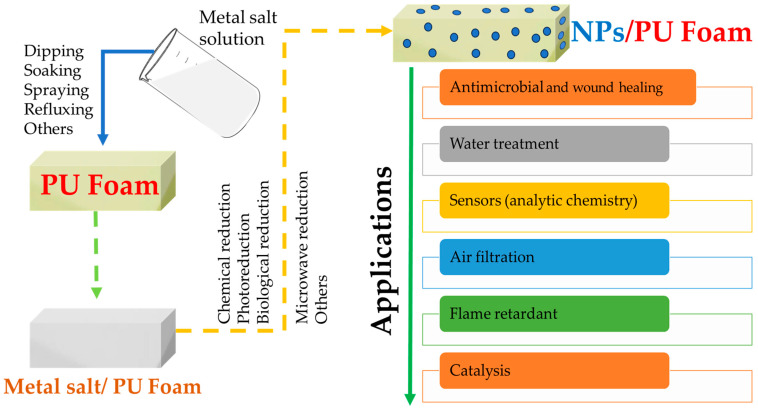
Schematic representation of NP/PU foam composite development using in situ-synthesized NPs and its applications.

Considering these aspects, examples regarding the incorporation of NPs into PU foams will be presented considering their synthesis route (ex situ—Table 2, schematically presented in Figure 3; in situ—Table 3, schematically presented in Figure 4).

Among the studies regarding ex situ-synthesized NPs, several present the use of commercially available nanoparticle dispersions [9,10,27,40,41,42,43]. The main advantage of this approach is a thorough control of the morphology of the nanoparticles, as well as the very good stability of NP solutions, which allows them to be used over long periods of time. Usually, this approach leads to a good dispersion of the nanoparticles in PU foams.

Regarding the NPs, Ag seems to be the primary choice for research in this area, either as metallic nanoparticles or as metal oxide NPs. The literature data present several examples of PU foams containing silver nanoparticles synthesized ex situ [9,10,13,21,27,28,40,41,42,43,44,45,46,47,48,49,50,51,52,53]. Besides antimicrobial applications (including water disinfection), the developed PU foam composites were tested for use as sensors, in electromagnetic shielding [49] and in pollutant removal [50,53]. Cu (either as a metal or as metal oxide nanoparticles), Zn (in its oxidized form) or silver ions or salts are also widely presented as PU foam reinforcements [12,14,54,55,56,57,58,59,60,61,62,63,64,65,66,67,68], either for antimicrobial applications [54,55,56,57,61,62,63,64], environmental protection applications [14,54,56,60,64,66,68], as flame retardants [57,65], in electromagnetic shielding [58] or other miscellaneous applications [59,67]. Other forms of ex situ-synthesized NPs, such as MgO, Au, W, TiO_2_, Pd, Fe_3_O_4_, Al_2_O_3_, SiO_2_ or Ni, have been explored in the literature for incorporation in PU foams [26,31,65,69,70,71,72,73,74,75,76,77,78]. Their application is usually connected with the NP’s individual properties, such as antimicrobial potential [31], and can be linked to catalytic or photocatalytic systems [26,71,77], pollutant adsorption [74,75], flame retardancy [65], shielding [72,73,78] or other industrial applications [76]. For example, multi-branched AuNPs, synthesized via ultrasound and chemical reduction (using hydroquinone), were evaluated by Huynh et al. [31] for incorporation in a classical PU foam to develop an antibacterial dressing. A minor influence of the NPs on the PU structure was exhibited in the developed material, with the foam retaining a relatively small average pore size (smallest dimensions: 98 nm). The intrinsic properties of more exotic NPs, such as W, can be applied for specific uses (such as a radiopaque agent), as demonstrated by Hasan et al. [69]. Their results demonstrated an increase in density with an increase in W incorporation (up to a maximum value of 0.060 g cm^−3^), as well as an increased viscosity, Young’s modulus and tensile strength (up to 4%W). At the same time, the foam maintained a constant overall porosity and demonstrated longer actuation times with an increase in W. Iron-oxide-NP-reinforced PU foams can find applications in environmental protection [74,75] or shielding [72,73].

Considering all these potential applications, reinforcement of PU foams with metal or metal oxide NPs can definitively be viewed as a viable approach, making use of their specific properties. However, a particular question arises when modifying an already established material: how are its properties influenced? Fortunately, several studies can provide satisfactory answers.

Several studies (see Table 2) suggest that the incorporation of NPs does not alter the open cell structure of the foam but provides a higher surface area and a smaller pore size (important elements for several applications), preserving or enhancing the foam’s mechanical properties. The only significant exception is represented by the study of Khan et al. [76], that revealed, for Al_2_O_3_ concentrations above 2%, significant alterations of the PU’s structure and properties. This is not, however, a surprise, as most authors define an optimal NP concentration (dependent on the NPs and incorporation route) above which the properties start to decline. Generally speaking, at concentrations under 2% NPs, the foams exhibited pore sizes in the range of 100–500 µm (which reduced with the addition of NPs compared to pure PU foams) and an increase in the compression strength and stability.

Sportelli et al. [12] evaluated the incorporation of CuNPs (obtained by the sacrificial-anode electrochemical method) into different types of PU foams (for mattresses and for the automotive industry) by the dipping method in order to develop antimicrobial foams. Their results suggested that the NPs do not affect the pore characteristics, while NP release is favored at a higher initial copper concentration and by the characteristics of the foams (foams with larger pores lead to higher and faster NP release).

Namviriyachote et al. [9] presented the incorporation of different concentrations of AgNPs, together with a herbal compound from *Centella asiatica* active in wound healing (asiaticoside), in PU foams based on natural polyols (hydroxypropyl methylcellulose, chitosan and sodium alginate) to develop a foam dressing for wound healing. The composite’s properties were more related to the type and content of natural polyols than to the NP content; the authors determined an optimal composition for both NP and herbal compound release. The study is important considering the alternative to classical chemical polyols, which opens another important research area. Another alternative for conventional organic-solvent-based polyurethane is represented by waterborne polyurethane. Zhao et al. [40] presented the incorporation of commercial AgNPs (15–40 nm) into waterborne PU foams via mechanical foaming for use as a bacteriostatic agent. Their results revealed the preservation of the open cell structure, with uniformly dispersed NPs. The optimal NP concentration identified by the authors was 2%, at which increases in the pore size, air permeability, water vapor transmission, and thermal and mechanical properties were recorded. Above this concentration, most of the mechanical and physical properties started to decline.

Phytosynthesized AgNP incorporation in PU foams was presented by Morena et al. [13]. The phytosynthesis process was applied using phenolated lignin under ultrasound irradiation, leading to nanoparticles with an average diameter of 13.29 nm. Impregnation was performed by dispersing the NPs in a polyol mixture at different concentrations. The addition of NPs preserved the open cell structure and led to a decrease in cell diameter. The optimal NP concentration that led to the highest compression modulus and swelling ratio increase was 0.12%NPs.

A very interesting study is represented by the work of Cheng et al. [21]. Although it is more focused on the antibacterial effect of the TiO_2_/Ag/chitosan embedded in the PU foams (an aspect that will be detailed in the next section) than on the NP’s characteristics and their influence on the final PU properties, the authors conducted a patient feedback study on a pain-reducing mattress constructed using the composites, with >85% of the responses evaluating it as “excellent”. This study offers a glimpse into the possible future applications of the solutions offered at the laboratory level by other works.

Another important application of PU foams is in electromagnetic interference shielding. Selvaraj et al. [78] presented the incorporation of a mixture of commercial NPs (MgO 40–60 nm, Ni 30–50 nm) in a bio-based PU foam via the dipping method. NP incorporation led to a potentially biodegradable, inexpensive, lightweight and flexible shielding material (maximum shielding: 27.56 dB).

Another metal nanoparticle which was evaluated for incorporation in PU foams not commonly encountered in the literature is Pd. Sahoo et al. [71] presented the incorporation of PdNPs obtained via hydrothermal synthesis using PVP as a coating material in commercially available PU foams via dipping. The authors evaluated the efficiency of the developed material as a recyclable catalyst for Suzuki−Miyaura cross-coupling reactions; their findings supported their potential application (the catalysts were viable after 50 catalytic cycles). More importantly, considering the goal of the present review, the authors also evaluated the penetration depth of the NPs (0.1 cm) and the morphology of the evaluated foam, revealing a structure comprising 3D interconnected 100−500 μm pores. The incorporation of SiO_2_ nanoparticles could also increase the mechanical durability and stability of the foams by providing water and oil repellency characteristics, as demonstrated by Cho et al. [77].

**Table 2:** Incorporation of metal and metal oxide nanoparticles formed ex situ into PU foams and the main non-antimicrobial findings; references are grouped considering the NP type ^1^.

NP Type	NP Synthesis Method and Characteristics	PU Foams	Synthesis Process	Application	Main Findings	Ref.
Ag	Commercial NPs	PU foams containing natural polyols (hydroxypropyl methylcellulose, chitosan and sodium alginate)	Active ingredients (AgNPs at 0.4, 0.6, 0.8 and 1.0 mg/cm^2^ and asiaticoside powder at 5%) adsorbed	Dermal wound dressing	Average pore size: 228–262 μm, viscosity slightly increased; higher concentration of polyols led to higher AgNP-releasing profiles. Optimal formulation: 6% natural polyols and 1 mg/cm^2^ AgNPs	[9]
Ag	Commercial AgNPs, PVP-coated, 100 nm diameter, surface area 5.0 m^2^/g.	Commercial flexible foam	Incorporation via mechanical stirring at different concentrations (0.1, 0.5, 1%)	Antimicrobial applications	Homogeneous dispersion of AgNPs in a polymeric matrix at low concentrations, cluster formation at higher loadings. Optimum concentration by step compression stress relaxation was 0.1% AgNP; resilience, hardness and compression unaltered by NPs. No change in thermal stability induced by NPs	[10]
Ag	Phytosynthesis using phenolated lignin and sonication, 13.29 nm (TEM)	PU formulation: PEG, DC 5179 additive, MDI, DABCO	Impregnation via NP dispersion in a polyol mixture at 0.12, 0.2, 0.25% relative to final composition	Chronic wound treatment	Open cell structure, cell diameter decreased with an NP concentration increase, 40% increase in compression modulus, swelling ratios varied from 585% (0.25% NPs) to 1145% (0.12% NPs), density increased with NP content	[13]
Ag	Commercial, 100 nm	Polypropylene glycol-based PU foam	Incorporation of NPs into a polyol mixture, foams converted to negative Poisson’s ratio or auxetic polyurethane	Medical cushioning	Foams made using a higher compression ratio exhibited an increase in compression strength at higher strains and a higher density compared to PU foam.	[27]
Ag/Ag_2_O	Mixture of NPs obtained by chitosan treatment, spherical, 44–75 nm (SEM)	Commercial PU foams	Impregnated with nanochitosan and nanosilver/silver oxide	Coliform removal from water sources	Increased surface area (2.17 m^2^/g)	[28]
Ag	NPs obtained by the borohydride technique—average diameter 19 nm	Polyether type polyurethane foam (commercial)	Immersion in NP solution	Potential applications in analytical chemistry	Materials stable for at least four days; uniform color, indicating satisfactory dispersion of NPs.	[44]
Ag	NPs obtained by reduction with chitosan, spherical shape and a size range of~50 nm	Commercially available	Dipping in AgNP solution	In vivo antibacterial study	Coating thickness 3–5 µm; PU coating average pore size 400–600 µm	[45]
Ag	NPs obtained by reduction with sodium citrate, 25 nm crystallite (XRD),	Obtained from commercial polyisocyanate and polyol reactants	Dipping in AgNPs for 2, 4, 6, 8 h	Antimicrobial applications (water disinfection)	No detectable agglomeration of AgNPs; total size and porosity of foam unaltered; AgNPs unwashed after repeated cycles	[46]
Ag	Synthesis by electric explosion of wire in liquid, 90 nm diameter (SEM)	Obtained from commercial polyisocyanate and polyol (ethylene oxide/propylene oxide random copolymer (polyethylene glycol)) reactants	Incorporation of AgNP and recombinant human epidermal growth factor in foams	Dressing material for biomedical applications	No influence of incorporation on PU foam surface pore size (200–400 µm), AgNPs embedded inside the pores	[47]
Ag	Fungi extracellular synthesis of NPs, 4.24–23.2 nm diameter for *Scopulaiopsos brumptii Salvanet-Duval* particles, 6–26 nm for *Peniciillium Citreonigum Dierck* particles, spherical morphology (TEM)	Commercially available	Incorporated by soaking over night	Removal of pathogenic bacteria from wastewater	No evaluation of the material’s characteristics	[48]
Ag	Commercial NPs, 15–40 nm	Waterborne PU foams (commercially available emulsion)	Incorporation by mechanical foaming, AgNPs—0–4%	Bacteriostatic agent	Open cell structure; NPs uniformly dispersed; aggregation at higher NP concentration a rougher surface; up to 25.8 nm (AFM), pore size increased up to 34.24 µm; improved thermal properties; increase in air permeability, water vapor transmission, tensile strength (up to 1.26 Mpa, 412.39% increase at 2% NPs); elongation at break decreased with AgNP addition	[40]
Ag	Polymer-template-assisted assembly using glucose, PVP and NaCl, 180 °C for 18 h	Obtained from commercial isocyanate and polyethylene polyol reactants	Impregnation with graphene oxide (7 mg/mL), carbon nanotubes (7 mg/mL), AgNPs (15 mg/mL) and dopamine (0.1 mg/mL)	Industrial applications (such as sensors and electromagnetic shielding)	Final composites reached 12.28 N/mm (tensile strength), improved thermal stability, electric conductivity properties (2 × 10^−4^ S/cm^−1^)	[49]
Ag	Phytosynthesized using hibiscus leaf extract, spherical, 50–70 nm (TEM), compared with commercial NPs	Commercially available (modified by chemical treatment—hydrophilic)	Surface-coated on polyurethane foam, or fused on polyurethane foam	Pesticide adsorption in column studies	Highest pesticide removal (96% at 20 mL/h) for fused polyurethane foam with commercial NPs, surface-coated polyurethane foam (CPU) and fused polyurethane foam	[50]
Ag	Commercial	Commercial formulations	Incorporation in PU foams, comparison with other biocidal additives	Biocidal applications	AgNPs had the least effect on the technological parameters	[41]
Ag	Commercial nanowires, 70 nm diameter, 100–200 µm length	Commercially available	NW solution sprayed over PU foams	Clinical wound healing	Composites revealed excellent elasticity without plastic deformation, hydrophobic character	[42]
Ag	Reduction with ethanol on the surface of natural zeolite, diameter 4.61 nm (TEM)	Open-cell soft polyurethane foam	NPs/zeolite mixed during PU production	Biocidal application	Open cell foam structure, mean cell size distribution 121.68 μm	[51]
Ag	Commercial NPs, 30 nm	Disocyanate and polyol PU foam	AgNP and AgNP/GO nanocomposites prepared by pepsin reduction mixed in the polyol solution	Antibacterial applications	Compared with AgNP loading, the use of AgNPs/GO led to a more homogenous dispersion, 1.85% resilience improvement, 7.9% tensile strength improvement, 6.52% tensile elongation at break improvement	[43]
Ag	Phytosynthesized using a *Verbena officinalis* leaf extract, 42.57 nm (SEM)	Obtained from commercial polyisocyanate and polyol reactants	Incorporation by mixing in polyol solution	Antimicrobial nano-biofilter	The number of foam cavities increased with addition of NPs	[52]
Ag/Ag_2_O	Mixture of NPs obtained by chitosan treatment, spherical, 44–75 nm (SEM)	Commercially available	Impregnation by dipping with nanochitosan, nanosilver/silver oxide and nanochitosan-nanosilver/silver oxide	Phosphate removal from water sources	Increase in surface area, superior sorption capacity compared to individual nano-components	[74]
Ag/TiO_2_	Produced by sintering at 600 °C, particle size 958.3 nm, by adding Ag to TiO_2_ NPs produced by sol–gel	Produced by the group, no recipe disclosed	Ag/TiO_2_/chitosan powder coated on bendable double mattress with added HAP powder	Bending mattress for bedridden patients	Bed mattress tested using a patient survey with good feedback	[21]
Cu	Sacrificial-anode electrochemical synthesis and TOAC stabilization, 2.6 nm diameter (TEM)	Commercially available. Two types of industrial foams, a filling material for mattresses (large and irregular pores, density: 25 kg/m^3^) and an automotive industry foam (small and regular pores, density: 21 kg/m^3^)	Dipping in diluted CuNP solutions (1:100, 1:1000)	Antimicrobial applications	Pore characteristics are not affected by NP uptake; higher and faster Cu release for higher initial CuNP solution and PU foams with larger pores	[12]
Cu	Nanosheets obtained via a CBD process	Commercially available	Dipping	Adsorption and antimicrobial properties	Pore size 150–500 μm, adsorption capacity 76.5 mg/g for Cr (VI), 714 mg/g for Congo Red dye	[54]
CuO	Direct thermal decomposition method, spherical shaped, 47.5 nm diameter (TEM)	Foams obtained via the one-shot method using a toluene diisocyanate and polyol system	CuONPs, starch and silicone surfactant mixed with polyol components	Antiseptic polyurethane foam dressings	Optimal NP synthesis at 600 °C, with optimal open cells of the corresponding foams	[55]
Ag_3_PO_4_	Precipitation	Obtained from commercial toluene diisocyanate, polyols and polyvinyl alcohol	Dispersed in a flexible open-cell polyurethane mixture, followed by graphene oxide coating	Antimicrobial properties and acid red 87 dye adsorption	Open cell structure, adsorption efficiency of 97% for 0.05 g of nanocomposite	[56]
Ag	Ions from AgNO_3_	Commercially available (30 kg/m^3^ density)	Successively dipping in poly(acrylic) acid, chitosan, Ti_3_C_2_ and metal solution	Flame retardancy and antibacterial applications	No visible damage; reduced thermal degradation rate, burning rate (156 mm/min, control 237 mm/min), PHRR, heat production speed; smoke suppression ability. Increased compression strength by 79.6%	[57]
Cu	Ions from CuSO_4_				Discontinuous coating: micro-cracking; no influence on thermal stability; reduced burning rate (208 mm/min, control 237 mm/min), PHRR, heat production speed; smoke suppression ability; increased compression strength by 38.4%	[57]
Cu	Electroless deposition	Thermoplastic polyurethane (TPU) granules, commercially available	Deposition on TPU/ANF/Ti3C2Tx Mxene	Detection of human motion and electromagnetic interference shielding	Board compressive interval (0–344.5 kPa, 50% strain), good sensitivity at 0.46 kPa^−1^, rapid response/recovery time (100 ms), electromagnetic interference shielding at 79.09 dB in X band	[58]
CuO	Arc discharge in a controlled atmosphere synthesis, spherical, average size 34 nm (TEM)	Rigid polyurethane foams obtained using high-molecular-weight tannins (from *Pinus radiata* bark), polymeric diphenylmethane diisocyanate, dimethyl sulfoxide, SoudaFoam FR, polyol	Mixing in polyol solution, final concentration 2%	Miscellaneous applications	Decreased pore size, strengthened cell walls, improved mechanical properties, elastic modulus (3.7 MPa) and stress (max. 1.13 MPa), apparent density	[59]
Zn	Chemical precipitation from commercial ZnO NPs	Commercially available	Dipping	Oil–water separation	Superhydrophilic/superoleophobic features (oil contact angle 158°, water contact angle 0°), oil separation efficiency up to 99.5%	[60]
ZnO	Sol–gel method, spherical, 40 nm diameter (XRD, TEM)	Obtained from commercial isocyanate and polyol reactants	Incorporation by mixing in polyol solution	Photocatalytic degradation of textile dye methylene blue	Increased density with NP content, reduction in cell diameter, increased exposed surface area, open cell structure, superior MB degradation under solar irradiation	[14]
ZnO	NPs obtained by the sonochemical method in a biopolymer (starch, gelatin, chitosan, and agar) matrix; crystallite sizes: 15, 26, 19, and 12 nm (XRD), average diameter 80, 41, 38, and 60 nm (TEM); morphology: microspherical/rice-like/nanospherical/egg-shaped	Furniture-grade polyurethane foam, commercially available	Coating with ZnO—biopolymer	Antifungal pillow materials for automobile and hospital industries	ZnO starch and ZnO chitosan—homogeneous adhesion spread through the foam walls, maintaining the softness of the foam; ZnO gelatin and ZnO agar—continuous film-like growth; all samples revealed UV photoactivity	[61]
ZnO	Precipitation method, spherical, 50 nm	Obtained from commercial isocyanate and polyol reactants	ZnO added in the polyol, followed by mechanical stirring, foams obtained by a two-step method	Antibacterial applications	Foams presented polygon closed-cell structures with energetically stable hexagonal and pentagonal faces, cell size comparable to unloaded foams, maximum tensile strength (193.5 kPa) and suitable compressive strength at 1.5% ZnONPs	[62]
ZnO	Commercial NPs, 50–250 nm	Obtained from diisocyanate and bio-based polyester polyol reactants	Incorporation of NPs by thermally induced phase separation at 1, 2, 5, 10%	Potential wound dressing	Flexible membranes, thickness 150–230 μm, similar porous structures, pore size 10–60 μm, small negative influence on thermal properties, increased hydrophobicity with NP content, lower absorptivity and acceptable WVTR (up to 8.9 mg/cm^2^·h)	[63]
ZnO	Chemical reduction, calcination, spherical, crystallite size 18.4 nm	Commercially available	PU foams refluxed with ZnO NPs for 6 h.	Antibacterial activity, detection and removal of basic dyes from wastewater	Detection limits of 2.5 and 2.9 μg/L for brilliant green and toluidine blue dyes, removal percentages of 92.4–98.2%, increased surface area, average pore radius of 3.4 nm	[64]
ZnO	Chemical synthesis using KOH, 20–80 nm (TEM), crystal size 27 nm (XRD)	Rigid polyurethane foam obtained from commercial isocyanate and polyol	NPs added to the foam mixture at 5% relative to polyol content	Flame-retardant rigid PU foam	Increased cell size, decreased density, pore diameter of 481 µm, lower burning velocity (346 mm/min, compared with blank—275 mm/min)	[65]
ZnO	Low-temperature chemical synthesis method, nanorods, 0.3 µm thickness, 1.2 µm length (SEM)	Commercially available	Multi-step dip-coating and seed-growth procedure	Photocatalytic treatment of aqueous acid red 88 dye	Highly porous, maximum color removal of 97% reached in 180 min under UVA	[66]
ZnO	Commercial NPs	Obtained from commercial isocyanate and polyol (castor oil derivative) reactants	NPs (6%) and sheath palm residues added during the polyol and isocyanate mixture	Miscellaneous applications	ZnO acted as a cell nucleation agent—homogeneous and isotropic cell structures. Increased resistance to heat absorption, thermal stability, foam crystallinity and stiffness	[67]
ZnO	Co-precipitation, crystallite size 15 nm (XRD), semi-regular spherical and rod-shaped structure.	Commercially available, apparent density 12–15 kg/m^3^ (97%)	Deposition on foam containing reduced GO by two successive impregnation and hydrothermal processes	Photocatalysts for methylene blue degradation	Good dispersion and embedment of NPs into the foam structure, ZnO NPs reduced the photodegradation capacity of PU foams containing reduced GO	[68]
MgO	Chemical synthesis using NaOH, 10–75 nm (TEM), crystal size 12 nm (XRD)	Rigid polyurethane foam obtained from commercial isocyanate and polyol	NPs added to the foam mixture at 5% relative to polyol content	Flame-retardant rigid PU foam	Increased cell size, decreased density, pore diameter of 514 µm, lower burning velocity (333 mm/min, compared with blank—275 mm/min)	[65]
Au	Multi-branched AuNPs, synthesized using hydroquinone as a reducing agent and chitosan as a stabilizer under ultrasound, 45 nm branches, 40 nm average size of core (TEM)	Obtained from commercial isocyanate and polyol	Dipping for 24 h	Antibacterial dressing	High water absorption, small average pore size (smallest dimensions 98 nm), 500% absorptivity	[31]
W	Commercial, 40–60 nm	Shape memory polymer foam obtained from isocyanate (NCO) pre-polymer and alcohols	WNPs dispersed in the NCO pre-polymer, prior to foam blowing at 4% to 11%.	Radiopaque agent for neurovascular occlusion applications	Density increased with W incorporation (up to 0.060 g cm^−3^); pore density and volume changed with loading, constant overall porosity; increased viscosity (with W addition), Young modulus and tensile strength (up to 4%W); longer actuation times with W increase	[69]
TiO_2_	Atanase form, hydrothermal treatment from tetrabutyl titanate and fluoric acid, 20–30 nm × 3 nm (TEM)	Commercially available	Dipping	Photocatalytic inactivation of airborne bacteria	Photoluminescence intensity decreases after loading with Mxene compared with pure TiO_2_	[26]
TiO_2_	Commercial spherical TiO_2_ (anatase), density 3.9 g/cm^3^, average diameter 25 nm	Commercially available flexible PU foams	Injection of NPs into the polyol followed by ultrasonic treatment	Industrial applications (sandwich panels)	Good dispersion of NPs in the matrix, decrease in cell size with NP content (up to 1%). 1% TiO_2_NPs foams—best thermal stability. Increased decomposition temperature, storage modulus, loss modulus and glass transition with NP addition	[70]
Pd	Hydrothermal synthesis using PVP, 2−6.5 nm (TEM)	Commercially available	Dipping	Recyclable catalyst for Suzuki–Miyaura cross-coupling reactions	NPs penetrated the foam up to 0.1 cm; foams contained 3D interconnected 100−500 μm pores; catalysts can be reused for 50 catalytic cycles	[71]
Fe_3_O_4_	Vacuum coprecipitation	Obtained from *Sapiumse biferum* kernel oil polyol and diphenylmethane diisocyanate	Incorporation in foam mixture	Lightweight renewable microwave-absorbing material	Porous structure; at 9% Fe_3_O_4_ content, foam exhibited microwave absorbency (effective bandwidth of 4.62/4.72 GHz at 1.789 mm/2.0 mm thickness in paraffin/bio-based polyurethane matrix). Effective absorbing frequency of 13.84 GHz at 5 mm thickness; saturation magnetization of 15.18 emu/g (superparamagnetism)	[72]
Fe_3_O_4_	Coprecipitation, 74 nm (TEM)	Obtained from commercial isocyanate and polyol	Incorporation of NPs and reduced GO by mixing in polyol solution	Electro-magnetic interference shielding material	Cylindrical cells with spherical shapes; average cell size of the composite decreases with filler concentration; cell density increased with the filler concentration; addition of the filler enhanced the compressive modulus and strength; maximum shielding efficiency 33 dB at 35% Fe_3_O_4_/rGO	[73]
Fe_3_O_4_	Commercial, 50–100 nm	Obtained from commercial isocyanate and polyol	Incorporation of Fe_3_O_4_@APTES (developed via sol–gel) by mixing in the polyol solution	Arsenic and heavy metal removal from water	Homogenous cell structure; higher surface area and lower pore size compared to PU (9.225 m^2^/g, 8.4 nm); removal efficiency of 95%, 86%, 79% for As/Cd/Zn.	[74]
Fe_3_O_4_	Phytosynthesis using *Simmondsia chinensis* (jojoba) defatted meal extract, rectangular shape 51.48 nm (XRD)	Commercially available	Impregnation through the dip adsorption method.	Drinking water defluorination	Increased thermal stability, superior adsorption capacity for Al_2_O_3_-modified foams (43.47 mg/g) compared to Fe_3_O_4_ (34.48 mg/g)	[75]
Al_2_O_3_	Phytosynthesis using *Simmondsia chinensis* (jojoba) defatted meal extract, irregular shapes 11.64 nm (XRD)	Commercially available	Impregnation through the dip adsorption method.	Drinking water defluorination		[75]
Al_2_O_3_	Commercial NPs, 40 nm (SEM)	Obtained from commercial isocyanate and polyol	Incorporation of Al_2_O_3_ at 1, 2, 3, 5, 10% by mixing in the polyol	Sandwich composites for industrial applications	Damage to cellular structure increased with NP content, lower glass transition temperature with NP increase, highest damping ratio and buckling peak for 2% NPs, decrease in stiffness and strength with addition of NPs	[76]
SiO_2_	Commercial, 20 nm	Commercially available (density of 30.3 kg/m^2^, tensile strength of 1.25 kg/m^2^, elongation of 130%)	Dip-coating	Bacterial anti-adhesion and antifouling applications	The foam demonstrated mechanical durability and stability; a high repellency to liquids such as water and oil; a high antifouling effect against polar and nonpolar liquid pollutants	[77]
MgO/Ni	Commercial nanoparticles, 40–60 nm/30–50 nm	Obtained from commercial isocyanate and bio-based polyol	Dipping	Electromagnetic interference shielding	Open cellular porous honeycomb morphology, average pore size: 300 μm, pore wall thickness: 15 μm, composites with 10% MgO and 1% Ni presented maximum shielding of 27.56 dB.	[78]

^1^ Abbreviations: NPs—nanoparticles, PEG—polyethylene glycol, MDI—4,4′-methylenebis(phenyl isocyanate), DABCO—1,4-diazabicyclo(2.2.2)octane, SEM—scanning electron microscopy, XRD—X-ray diffraction, TEM—transmission electron microscopy, NCO—isocyanate, TOAC—tetraoctylammonium chloride, AFM—atomic force microscopy, PVP—polyvinylpyrrolidone, WVTR—water vapor transmission rate, HAP—hydroxyapatite, MB—methylene blue, NW—nanowire, CBD—chemical bath deposition, GO—graphene oxide, PHRR—peak heat release rates, TPU—thermoplastic polyurethane, APTES—(3-Aminopropyl)triethoxysilane.

The second major alternative for the development of NPs containing PU foams is represented by the in situ formation of the nanoparticles in the PU matrix (Figure 4).

Although not as common as the ex situ formation, the literature data provide several examples regarding the development of PU foams containing in situ-synthesized NPs, including Ag [7,8,44,79,80,81], Cu [82], ZnO [83], SiO_2_ [84], FeOOH [85], MnO_2_ [86] or mono- and bi-metallic noble metal NPs [87].

Silver nanoparticles are commonly obtained in the PU matrix either by chemical methods or by photoreduction. Their potential applications vary from wound healing and antimicrobial applications [7,8,80,81] to analytical applications [44] or air filtration systems [79]. An interesting study is represented by the work of Apyari et al. [44], who comparatively evaluated both methods for incorporation of AgNPs. The reduction of sorbed silver nitrate was achieved by the use of ascorbic acid, and the authors established the optimal conditions as a reaction medium of 0.05 M sulfuric acid and a reaction time of 40 min. Besides these results, the authors concluded that the material obtained was more promising for applications in analytical chemistry for the determination of oxidants and reductants compared with the composite obtained using ex situ-synthesized NPs.

Li et al. [82] evaluated the antimicrobial applications of CuNP/PU foams, as well as the morphology and mechanical characteristics of the developed materials. The authors determined that the cell structure was not significantly influenced by the development of NPs, while the open cell content decreased from 97.42 to 96.64%, accompanied by tensile and compressive strength improvements, thus recommending the materials for antimicrobial and water treatment applications.

An important issue related to the widespread use of PU foams is their highly combustible nature. A study by Meng et al. [85] could provide an alternative solution to this issue. The authors incorporated FeOOH NPs obtained via chemical precipitation on PU foams through in situ surface growth in order to develop an antimicrobial flame-retardant coating. The multifunctional PU foam composite exhibited a limiting oxygen index of 25.5%, a reduction in the peak heat release rate of 45.0% and in the smoke density of 69.1% and a good underwater superoleophobicity.

A more complex study was recently published by Gazil et al. [87], evaluating the in situ development of mono- (Au, Ag, Pd) and bi-metallic (AuPd) nanoparticles in PU foams via microwave irradiation and hydrothermal synthesis for their application as catalytic sponges in semiautomated synthesis. Their conclusions were that the mono-metallic NPs obtained an open cell structure, with smooth surfaces of the cell walls and a homogeneous distribution of nanoparticles on cell walls. Regarding their potential applications, the reaction rate obtained using the materials was comparable to state-of-the-art catalysts. At the same time, for the bimetallic nanoparticles, the open cell structure was preserved; however, the NPs had an inhomogeneous distribution and morphology, while the obtained reaction rates for 4-nitrophenol reduction were inconsistent.

**Table 3:** Incorporation of metal and metal oxide nanoparticles formed in situ into PU foams and main non-antimicrobial findings; references are grouped considering the NP type ^1^.

NP Type	NP Synthesis Method and Characteristics	PU Foams	Synthesis Process	Application	Main Findings	Ref.
Ag	In situ formation, quasispheric, 20–30 nm (TEM)	PU constructed using lignin-based polyols	Dipping in metal salt solution	Wound healing applications	Porous morphology, average pore diameter: 100 μm, pore size decreased with NP concentration increase, improved residual ash content, initial degradation temperature and mechanical strength, best results obtained for the highest Ag concentration	[7]
Ag	Spherical nanoparticles, 6–10 nm (TEM), smaller dimensions for inner particles	Commercial open-cell PU foam (average density 18.5 g dm^−3^)	Intermatrix synthesis inside foam via the NaBH_4_ method	Catalytic and bactericidal water treatment	Up to ten times higher metal content uptake compared with ex situ formation; stable final composites (<1% Ag leaching); significant catalytic activity, not diminished after 3 cycles	[8]
Ag	Average diameter 52 nm (SEM)	Polyether-type polyurethane foam (commercial)	In situ reduction of silver to NPs using ascorbic acid	Potential applications in analytical chemistry	In situ optimal synthesis—0.05 M sulfuric acid, 40 min.	[44]
Ag	Photoreduction (UV) synthesis of NPs, spheroidal NPs, grouped in 150–200 nm clusters	Industrial PU foams	Direct synthesis on the foam	Filters for air filtration	AgNPs penetrated the foam up to 5 mm with good homogeneity, no altering of the porous structure or polymeric surface chemical composition, fast release of antibacterial ions in physiological solution	[79]
Ag	Chemical synthesis (using NaBH_4_ method) and phytosynthesis using neem leaf extract	Obtained from commercial isocyanate and polyols (castor oil) reactants	Inter matrix synthesis approach, reduction performed directly on silver impregnated PU foams	Biomedical applications	NPs enhanced PU thermoxidative degradation (lower degradation temperature)	[80]
Ag	In situ reduction	Commercially available (76 par per inch, density 30.4 kg/m^3^)	Reduction of Ag^+^ ions to form AgNPs with glycerol in calcium alginate (CA)/PU foam composite	Antibacterial agent for point-of-use water disinfection	CA/PUF@Ag composites prepared with 0.25% *w*/*v* CA present a higher swelling ratio (8.0 g/g), larger initial AgNP loading (8.48 mg/g), a lower Ag release concentration (44.35 μg/L) and a lower Ag release rate (0.27%) after 14 days	[81]
Cu	In situ generated, at different copper concentrations	Neat flexible PU foams obtained by a one-step process	Dipping in CuSO_4_, maintained at 80 °C	Antimicrobial applications	Cell structure not significantly influenced; open cell content decreased from 97.42 to 96.64%; tensile and compressive strength improved, respectively, from 78.1 to 94.2 kPa and from 3.80 to 5.63 kPa	[82]
ZnO	Hydrothermal synthesis by seeding on the surface of PU foams	Commercially available	Dipping	Photodegradation of acid black 1 dye under UV and solar light	85%/65% dye degradation achieved under UV/solar light irradiation.	[83]
SiO_2_	Sol–gel synthesis in PU matrix, 5–60 nm	Obtained from commercial isocyanate and polyols reactants	Direct synthesis of the hybrid foams	Biomedical applications (dressing foams)	Low structural integrity of foams at >10% Si; increased stiffness with the silica contents; significant increase in durability, strength and elongation; no significant change in water vapor transmission rate	[84]
FeOOH	Chemical precipitation	Commercially available (polyether-based polyurethane foam, density, 30.00 kg/m^3^)	In situ growth on the surface of PU foam containing oxidized sodium alginate and dopamine	Flame-retardant coating, antimicrobial applications	LOI reached 25.5%, peak heat release rate reduced by 45.0%, smoke density decreased by 69.1%; good underwater superoleophobicity (oil contact angle under water 155.2°)	[85]
MnO_2_	In situ formation	White and color industrial polyurethane foam waste from various sources: scrap from households, upholstery stores, furniture factories	Refluxed with KMnO_4_ in acidic medium	Antibacterial applications and removal of anionic and cationic dyes	MnO_2_ randomly distributed inside the spaces of the matrix, paramagnetic behavior (2.5 × 10^−5^ erg/G^2^ g), superior surface area (14.3 m^2^/g), 97.5–100% removal of methylene blue dye, 85–87% removal of Trypan blue	[86]
Au	Microwave irradiation, hydrothermal synthesis, 30 nm, seeds of 3 nm (TEM)	Commercial PU foam	In situ synthesis, foam inserted into the reaction medium	Catalytic sponge for semiautomated synthesis	Open cell structure, smooth surfaces of the cell walls, nanosized particles homogeneously distributed on cell walls, reaction rate comparable with state-of-the-art catalysts	[87]
Ag	Microwave irradiation, hydrothermal synthesis, 16 nm (TEM)					
Pd	Microwave irradiation, hydrothermal synthesis, 5 nm (TEM)					
AuPd	Microwave irradiation, hydrothermal synthesis, Au—130 nm, Pd—6 nm (TEM)				Open cell structure, smooth surfaces of the cell walls, nanosized inhomogeneous particles, inconsistent reaction rates for 4-nitrophenol reduction	

^1^ Abbreviations: NPs—nanoparticles, SEM—scanning electron microscopy, TEM—transmission electron microscopy, LOI—limiting oxygen index.

All these examples, alongside the other studies presented in Table 2 and Table 3, reveal the possibilities of NP incorporation in PU foams, as well as the many different areas in which the developed composites can find applications.

## 6. Antimicrobial Properties and Biocompatibility of Metal-Nanoparticle-Modified PU Foams

As presented in the previous section, most works have highlighted the antimicrobial properties of the developed materials. Therefore, the present section provides the main findings regarding the antimicrobial efficiency reported within these studies, summarized in Table 4.

Among all metals, silver-based antibacterial agents are the most widely studied and applied. Although their mechanism of action on microorganisms has not been entirely elucidated, various hypotheses have been proposed. The main antibacterial actions include the continuous release of silver ions, their adherence to the bacterial cell wall and membrane (permeabilization or disruption of the cytoplasmic membrane), oxidative stress induction and modification of signal transduction pathways [95]. The accumulation of Ag NPs on the cell surface was observed especially in the case of Gram-negative bacteria. The absence of a thick cell wall and the presence of negatively charged lipopolysaccharides make them much more sensitive to the action of Ag NPs than Gram-positive bacteria [96]. Once inside the cell, metal ions can interfere with the signaling pathways of bacterial metabolism and growth by inhibiting ATP production and the synthesis of proteins involved in cell viability and division [46]. However, along with their antibacterial properties, metal and metal nanoparticles also have a series of adverse effects on the environment and human health. For example, silver exhibits some potential toxic effects in aquatic ecosystems and against human-friendly soil microbial communities (such as nitrogen-fixing and ammonifying bacteria) [97].

Metal nanoparticles can enter ecosystems through various pathways, such as industrial runoff or the disposal of products containing these nanoparticles. Once released into the environment, they can accumulate in the soil and water, leading to bioaccumulation in plants and animals, potentially causing adverse effects to ecosystem health [98,99,100]. Furthermore, these nanoparticles may undergo transformations in the environment, altering their chemical and physical properties and potentially enhancing their toxicity. Their small size and increased surface area can also facilitate their transport over long distances, increasing the risk of widespread environmental contamination [101].

To address these concerns, in addition, there are several reports that describe the biocompatibility of these metal-nanoparticle-modified PU foams with animal cells, exhibiting an efficient wound healing activity. In this way, silver nanoparticles were included in CuraVAC Ag, a device that administers negative pressure wound therapy through a polyurethane foam dressing and discharges ions onto a wound surface where they are saturated with water, providing high efficiency scores of wound healings on rats [45]. Also, lignin-based PU foams with silver nanoparticles were applied to full-thickness skin wounds on mice, demonstrating higher wound healing abilities than Tegaderm film, as demonstrated by well-proliferated granulation tissue formation, re-epithelialization, angiogenesis and dense collagen deposition [7].

Besides animal studies, there are also several reports on in vitro cytotoxicity assessments of PU foams in order to confirm their benefits for the management of burns, limiting the number of suffering animals. Boonkaew et al. showed that their own developed hydrogel dressing based on a 2-acrylamido-2-methylpropane sulfonic acid sodium salt and silver nanoparticles had lower toxicity to human keratinocytes (immortal cell line HaCaT and primary cells HEK) and fibroblasts (NHF) compared to commercially available silver products (Acticoat^TM^ and Flamazine^TM^) after 24 h of incubation in Nunc^TM^ polycarbonate inserts [102]. No cytotoxicity to HaCaT cells and BJ5ta fibroblasts was also observed for PU foams with incorporated lignin-capped silver nanoparticles, which had radical-scavenging activity and an ability to reduce the ex vivo myeloperoxidase activity in wound exudate [13]. Another cell line used to confirm the cytocompatibility is the 3T3 murine fibroblast line, as reported by Picca et al. for Ag-modified PU foams [79]. Further, human-adipose-derived stem cells (hASCs) were cultivated in direct contact with TPU/ZnO nanocomposite foams, displaying the highest cell proliferation for 2 and 5 wt% ZnO [63]. This type of cell was also used by Norozi et al. to demonstrate the ability of PU foams with ZnO to help cellular adhesion, proliferation and osteogenic differentiation [103]. Mouse embryonic fibroblasts are another type of cell used to test the biocompatibility of PU foams, as in the case of those incorporated with nanosized copper-benzene-1,3,5-tricarboxylate [90].

Novel synergistic dressings, comprising silver nanoparticles and recombinant human epidermal growth factor in PU foams, provided a good cytocompatibility with mice fibroblasts L929 and significantly accelerated the healing of diabetic wounds, with complete re-epithelialization in a diabetic BALB/c mice model [47]. Full-thickness wounds treated with PU foams with 5–60 nm silica nanoparticles (with a content of up to 10 wt%) demonstrated accelerated wound closure rates in Sprague-Dawley rats and collagen and elastin fiber regeneration in a newly formed dermis covered by a new epithelial layer [84]. In addition, these PU-Si hybrid foams promoted L929 cell proliferation to a greater extent than pure PU (*p* < 0.05) [84].

A PU foam dressing containing 6% alginate, 1 mg/cm^2^ silver and 5% asiaticoside proved its lack of cytotoxicity on human skin fibroblasts (BJ cell line), lack of skin irritation in rabbits and improved wound healing in pigs without any dermatologic reactions [9].

A silver-nanowire-based 3D porous foam dressing developed by Chen et al. [42] showed high levels of live cells after 48 h of incubation with 3T3 cells and promoted wound healing in Bama pigs in combination with exogenous electric fields. This combined therapeutic system facilitated necrotic tissue drainage, downregulated the expression of E-cadherin to weaken intercellular adhesion and promoted angiogenesis, cell migration and proliferation.

## 7. Other Types of Modified PU Foams with Antimicrobial Properties

As previously mentioned, the main goal of the presented review is a review of the current state of the art regarding incorporation of metal and metal oxide nanoparticles into PU foams (especially for biomedical and related applications). In order to provide an image of the possibilities in the development of antimicrobial PU foams, the following section will provide a brief presentation of the progress in the last few years regarding other antimicrobial nanomaterials incorporated into PU (summarized in Table 5). Besides the efficiency of inhibiting bacterial growth, several PU foams have been designed for skin applications, exhibiting a good biocompatibility and cell adherence in in vitro studies [104,105,106] and improved wound healing in in vivo murine models [104,107]. These types of materials are important to briefly present, as they can be considered as a starting point for future research.

As can be seen from the examples provided in Table 5, PU foams can be easily modified with a large variety of other types of materials, although the impregnation/reinforcement routes are similar to those described in previous sections. Also, if, for simpler fillers, the addition can be performed via incorporation into the polyol mixture (without a negative effect on the foam structure and characteristics), for more complex fillers, a deposition strategy should be adopted. The latter case (especially when the reinforcement material forms a second layer and does not diffuse through the foam) raises some issues regarding the different properties of the layers. The issue can be overcome by carefully selecting the most appropriate coating for the PU foam.

All the above examples underline the great potential of modified PU foams, especially regarding antimicrobial applications.

## 8. Conclusions and Future Perspectives

As emerging from the literature review, several synthesis methods for NPs were evaluated for reinforcing PU foams. Among those, green synthesis methods were relatively poorly studied, with biosynthesis (using fungi) and phytosynthesis (using natural extracts) being the only evaluated routes. The production processes for metal nanoparticles often involve energy-intensive methods and the use of hazardous chemicals [110], contributing to environmental pollution. The use of alternative nanomaterials, such as nanolignin, nanoclay, chitosan or montmorillonite (as presented in Section 7), could provide increased biocompatibility for skin applications, without posing an environmental threat. Further, the use of green synthesis methods for the development of nanoparticles can constitute a viable approach (some scarce literature data have already been presented on this topic for both ex situ [48,50,52,75] and in situ [80] routes); this leads to the proposal of other green synthesis routes, such as the use of ionizing-radiation-assisted synthesis, a method that our group previously used to provide small-dimension nanoparticles with good antimicrobial properties [111].

Regarding the characterization of PU foams, the existence of dedicated standards represents a tremendous advantage [112,113,114,115,116,117,118,119,120,121,122,123,124,125,126,127,128], although not all the studies presented followed their recommendations. In our opinion, these standards should be applied when evaluating the appropriateness of PU foams in order, on the one hand, to provide reproducible and comparable results, and, on the other hand, to demonstrate the market potential of the formulations.

Another very important aspect that can be considered in future research is represented by the replacement of conventional organic-solvent-based polyurethane by either waterborne polyurethane or bio-based polyurethane, as several authors have pointed out. Together with the adoption of green synthesis routes for NPs, this would lead not only to PU foams with superior mechanical and physical properties, adapted to the envisaged application, but also to more eco-friendly solutions.

## Figures and Tables

**Table 1:** Definition of the PICO strategy applied in the present work.

P (Problem)	Insufficient Antimicrobial Properties of PU Foams
I (Intervention)	Development of nanomaterial-containing PU foams for biomedical applications
C (Comparison)	PU foams, other antimicrobial materials
O (Outcome)	Development of antimicrobial PU foams containing metallic nanoparticles

**Table 4:** Antimicrobial and cytotoxic properties of metal-nanoparticle-modified PU foams; references are presented in chronological order ^1^.

NP Type	Metal Content, as Described by Each Study	PU Matrix	Microorganisms	Main Findings on Antibacterial Activity	Ref.
ZnO	0.2% biopolymer-ZnO (ZnO final concentration—40 mg/g)	Commercial PU foams coated with ZnO—functional biopolymers: starch, gelatin, chitosan, and agar (1:20 solid to liquid ratio)	*Aspergillus niger*	Optical microscopic images showed that ZnO containing agar coatings has no significant fungus growth	[45]
Ag	1.0% *w*/*w*	Medical-grade PU foam dipped in AgNPs	*S. aureus* *E. coli*	99.9% reduction in viable cell numbers after 1 h, 6 h, 12 h, 24 h, and 48 h of exposure	[52]
Ag	-	Commercial V.A.C. GranuFoam Silver Dressing	*S. aureus* *S. epidermidis*	ZOI: 4.4 mm after 24 h and 4.7 mm after 39 h ZOI: 7.5 mm after 24 h and 8.1 mm after 39 h	[88]
Ag	1.04 × 10^−3^ M (colloidal suspension)	PU foam soaked in a AgNP solution for 8 h	*E. coli*	Bacterial growth inhibition after 20 min exposure to polyurethane coated with AgNPs	[46]
Ag	0.1, 0.5 and 1%	PU foam incorporating AgNPs by mechanical stirring	*Klebsiella* sp. *Staphylococcus* sp. *E. coli*	Bacterial growth inhibition by 0.1% AgNP foam	[10]
Ag	11 to 21 mg Ag per g matrix	Commercial open-cell PU foams loaded with AgNPs obtained via intermatrix synthesis	*E. coli*	100% of bacteria killed in less than 6.5 h; the bacterial mortality rate was ca. 1000 CFU mL^−1^ s ^−1^	[8]
Cu	0.3–1.3%	Two types of industrial PU foams dipped in diluted CuNP solutions (1:100, 1:1000)	*S. aureus* *E. coli* *K. marxianus*	A higher CuNP loading was generally correlated to a higher concentration of released ions and an increased inhibition of colony growth after 24 h	[12]
Ag	-	PU foams incorporating AgNPs and recombinant human epidermal growth factor	*S. aureus* *E. coli*	ZOIs for AgNP-PUFs and AgNP/rhEGF-PUFs were significantly larger than that of the PUFs and at the same time higher against *S. aureus* after 24 h	[48]
Ag	1118.6 mg/L	PU foams soaked in AgNP solution overnight	Fecal coliforms Fecal streptococci *S. aureus*	Effective removal of total coliforms (97.3%), fecal coliforms (99.9%), fecal streptococci (99.9%) and *S. aureus* (99.9%) from wastewater after 24 h	[46]
Ag	100 g/m^2^	Direct synthesis of AgNPs on an industrial PU foam surface	*S. aureus* *E. coli*	96% and 97% bacterial reduction after 24 h; no bacterial growth was observed in the 24 h following the recultivation of surviving bacteria	[47]
Ag	0.95 mg/cm^2^ 1.20 mg/cm^2^ 0.50 mg/cm^2^ 0.90 mg/cm^2^ 1.34 mg/cm^2^ 1.30 mg/cm^2^	Commercial PU foam dressings: Biatain Ag, Mepilex Ag, UrgoCell Silver, Allevyn Ag, Acticoat Moisture Control Ag, PolyMem Silver	*E. coli* *S. aureus* *P. aeruginosa*	Biatain Ag, Mepilex Ag, and Allevyn Ag showed the highest antibacterial activity under challenging conditions with human acute wound fluid	[89]
CuO	-	Foams obtained via the one-shot method incorporating CuO NPs, starch and silicone surfactant mixed with polyol components	*E. coli* *S. aureus* *P. aeruginosa* *E. faecalis* *C. albicans*	The highest antimicrobial activity against hospital infections was obtained for CuO NPs obtained at 600 °C after only 120 min of exposure	[55]
ZnO	1.5 wt%	ZnO added to the polyol, followed by mechanical stirring, foams obtained by a two-step method	*E. coli* *S. aureus*	Bacterial growth reduction after 24 h with a more pronounced effect against *E. coli*	[62]
Ag	0.4, 0.6, 0.8 and 1.0 mg/cm^2^	AgNPs and asiaticoside powder at 5% adsorbed on PU foams containing natural polyols (hydroxypropyl methylcellulose, chitosan and sodium alginate)	*P. aeruginosa* *S. aureus* *E. coli* *B. subtilis*	Great antibacterial activity for PU formulations with 1 mg/cm^2^ silver (ZOI: ~ 2.5–3.5 mm)	[9]
Ag	0–4 wt%	Waterborne PU foams incorporating AgNPs via mechanical foaming	*E. coli* *S. aureus*	PU matrix filled with 2 wt% AgNPs proved its antibacterial activity (bacteriostatic rates were 98.23% and 97.38%, respectively)	[40]
ZnO	1, 2, 5, 10%	Thermoplastic PU foam incorporating ZnO NPs via the thermally induced phase separation method	*S. aureus* *E. faecalis* *E. coli* *P. aeruginosa*	The highest ZnO concentration (10%) led to a 10^3^ fold reduction in CFUs; 55% reduction in biofilm formation on the surface of the composites with no significant differences between ZnO concentrations	[63]
Ag	0.12, 0.2, 0.25% relative to final composition	Impregnation of NP dispersion in polyol mixture	*S. aureu* *P. aeruginosa*	An increase in the Ag NP content in the foams led to a higher antibacterial activity. PUF-0.25%NP showed over 4 and 5 logs bacterial growth reduction	[13]
Ag/TiO_2_	-	Ag/TiO_2_/chitosan powder coated on a bendable double mattress with added HAP powder	*S. aureus*	99% antibacterial efficiency	[21]
Ag	1 wt%	PU foam incorporating Ag NP/zeolite during production	*E. coli* *M. luteus*	More pronounced antibacterial effect against Gram-positive bacteria (*M. luteus*)	[51]
Ag	1 wt%	Foams converted to negative Poisson’s ratio or auxetic PU foam incorporating AgNPs	*S. aureus* *S. epidermidis* *P. aeruginosa* *E. coli*	A higher compression factor greatly enhanced the antibacterial activity	[27]
Ag_3_PO_4_	-	Ag_3_PO_4_ NPs dispersed in a flexible open-cell polyurethane mixture, followed by graphene oxide coating	*S. aureus* *E. coli*	0.1 g of GO/Ag_3_PO_4_ PU foam inhibited the colonies’ growth after 24 h	[56]
Cu	-	PU foam incorporated with Cu-BTC NPs	*P. aeruginosa* *K. pneumoniae* methicillin-resistant *S. aureus*	Selective and significant bactericidal effect; efficiency rates: 66.3% 99.3% 30.8%	[90]
Cu	0.2 M (colloidal suspension)	PU foam dip coated with Cu NPs	*E. coli* *S. aureus*	0.2 g of Cu PU foam effectively removed bacteria from wastewater in 3 h	[54]
TiO_2_	-	PU foams coated with {001}TiO_2_/Ti_3_C_2_T_x_ (MXene) nanosheets	*E. coli*	Superior inactivation efficiency of airborne *E. coli* under UV photocatalysis; Different inactivation mechanisms between UV irradiation and UV photocatalysis (bacteria are not able to reactivate after photocatalytic oxidation)	[26]
Ag	0.002, 0.021, and 0.088 wt%	PU foam obtained using lignin-based polyols dipped in metal salt solution	*E. coli* *S. aureus*	>99% antibacterial rate against *E. coli* within 1 h and *S. aureus* within 4 h	[7]
Ag	10% *w*/*w*	Commercial (GF Silver)	methicillin-resistant *S. aureus* *A. baumannii*	ZOI: 1.52 mm ZOI: 2.04 mm	[91]
Ag	50 and 100 mg	PU foams obtained by mixing “green” Ag NPs in polyol solution	*Y. ruckeri*	ZOI: 15.33 ± 1.6 for 50 mg Ag and 14.83 ±0.76 mm for 100 mg Ag	[52]
Ag	0.002 M (silver nitrate solution)	PU foams impregnated with AgNPs incorporated by intermatrix synthesis	*E. coli* *B. subtilis*	AgNP increased the ZOI diameter, showing antibacterial action against both bacterial strains	[80]
Ag	0.4%	AgNPs and AgNP/GO nanocomposites prepared by pepsin reduction mixed in the polyol solution	*S. aureus*	The foam containing AgNP/GO induced a larger ZOI, as it is a more effective antibacterial agent	[43]
Ag and Cu	1 wt%	PU foams successively dipped in poly(acrylic) acid, chitosan, Ti_3_C_2_ and metal solution	*P. aeruginosa* *S. aureus*	Significant reduction in bacterial growth (Ag-coated PU: 99.97% for *P. aeruginosa* and 88.9% for *S. aureus*; Cu-coated PU: 58.7% for *P. aeruginosa* and 72.4% for *S. aureus*)	[57]
Ag/AgO	-	PU foams impregnated with nanochitosan and Ag/AgO NPs	*E. coli*	100% removal efficiency	[28]
Cu	1 mM, 5 mM, 25 mM, 125 mM, 250 mM (copper salt solution)	PU dipped in CuSO_4_ solution	*E. coli* *P. aeroginosa* *B. licheniformis* *S. aureus*	Good antibacterial activities were obtained even with low concentrations of CuSO_4_ (ZOI varied between 28 and 40 mm)	[82]
Au	5–15 μg/mL	Au multi-branched NPs incorporated into PU foam by dipping for 24 h	*S. aureus* *E. coli*	>95% and ~ 85% removal efficiency	[31]
Ag	0.5 mM, 2 mM, 5 mM (silver salt concentration)	Calcium alginate (CA)/PU foam composite decorated with Ag NPs	*E. coli*	ZOI: 1.8–4.5 mm; OD600 value of the bacterial suspension filtered through CA/PUF@Ag decreased to a very low level (<0.05)	[81]
Ag	-	Commercial PU Foam–Ag Salt	*P. aeruginosa* *S. aureus*	Unable to inhibit bacterial biofilm	[92]
Cu	-	Nanosized Cu and graphene were incorporated into PU mix	*E. coli*	Viability of planktonic and adhered *E. coli* reduced to 99.66% and 96%	[93]
CuS	50, 100 and 150 μg/mL	Immobilization of CuS NPs on PU foam via the seeding method	*B. cereus* *P. aeruginosa*	Significant difference between bacterial strains (no antibacterial effect of the 50 μg/mL PU composite on *P. aeruginosa* growth); Excellent antibacterial activity for the highest content of CuS NPs (ZOI: 15 mm for *B. cereus* and 12 mm for *P. aeruginosa*)	[94]
FeOOH	-	In situ growth on the surface of a flexible PU foam containing oxidized sodium alginate and dopamine	*E. coli* *S. aureus*	Colonies of both bacteria did not grow on modified PU surfaces, and the number of both bacteria decreased significantly after 12 h	[85]
ZnO	1 wt%	PU foams refluxed with ZnO NPs for 6 h	*E. coli* *S. aureus* *S. typhimurium*	A decrease in bacterial growth was observed after 4 h; the antibacterial effect was more pronounced for *S. aureus* and *S. typhimurium*	[64]
SiO_2_	-	Fluorinated silica NP suspension deposited on PU foams via dip-coating	*E. coli* *S. epidermidis*	Reduction by >90% per unit area (1–2 log units) in bacterial adhesion	[77]
MnO_2_	-	PU foam wastes refluxed with KMnO_4_ in acidic medium	*B. cereus* *S. aureus* *E. coli*	ZOI: 8.8 mm ZOI: 7.5 mm ZOI: 7.1 mm	[86]

^1^ Abbreviations: NP—nanoparticle, ZOI—zone of inhibition, Cu-BTC—copper(II)-benzene-1,3,5-tricarboxylate; CFUs—colony forming units; OD—optical density.

**Table 5:** Antimicrobial properties of other types of nanomaterial-modified PU foams; references are presented in chronological order ^1^.

NM	PU Foam Composition	Assays	Main Morphological Findings	Main Findings on Biological Activity	Ref.
NL, 45–80 nm	Polyethylene glycol, glycerol, NL, 1, 6-diisocyanato-hexane (NCO/OH ratio: 1.2) and water as a blowing agent, coated with propolis	Morphological investigations Antimicrobial—ZOI test against *Staphylococcus aureus* (ATCC 25923) and *Escherichia coli* (ATCC 25922) Biocompatibility (L929 fibroblasts) In vivo wound healing	Increased tensile strength, and elongation at break; average pore diameter 110 µm, apparent porosity 87.9%, density 0.28 g/cm^3^_,_ water absorption 242%, contact angle 50.1 ± 2.1° ZOI: *E. coli* 7.2 mm, *S. aureus* 11.2 mm Cell viability > 90%, good fibroblast adhesion Significantly (*p* < 0.05) higher wound closure rate (~90% after 10 days) compared with the control (<60%)	Antibacterial activity (ZOI: *E. coli* 7.2 mm, *S. aureus* 11.2 mm); Good biocompatibility on L929 fibroblasts (cell viability > 90%, good cell adhesion); Significantly (*p* < 0.05) higher wound closure rate (~90% after 10 days) compared to control (<60%) in in vivo rat studies	[104]
NCl, Cloisite 30B	Hexamethylene diisocyanate, poly(ethylene glycol) reacted in tetrahydrofuran with tin(II) and added to a mixture of poly(glycerol sebacate) and Cloisite 30B; the resulting mixture was casted into polytetrafluoroethylene molds	Morphological drug loading and release tests. Biodegradation (lipase enzyme) Biocompatibility (L929 cell line)	Excellent transparency, pore size 94.3 µm, Young’s moduli 0.10 MPa, compressive stress at 75% strain values 0.29 MPa, contact angle 86.0°, water swelling ratio 212.3%; dye loading MB 41.8 mg/g, MO 15.6 mg/g, SG 6 mg/g, dye release 11.1/12.6/3.4 mg/g Mass loss with lipase 35.6% No evidence of cytotoxicity, increasing cell metabolic activity and good cell morphology	No evidence of cytotoxicity on L929 fibroblasts, increased cell metabolic activity and good cell morphology	[105]
Composite: poly(ethylenimine), poly(acrylic acid), Na^+^ montmorillonite, poly(diallydimethylammonium chloride), chitosan and sodium alginate	Composite deposited via the layer-by-layer technique on commercial PU foams	Morphological and mechanical assays Cell viability	Open cell structure, elastic modulus increased up to 6.01 MPa, similar porosity Significantly lower cytotoxicity for the chitosan and poly(diallydimethylammonium chloride) coatings	Significantly lower cytotoxicity for the chitosan and poly(diallydimethylammonium chloride) coatings on U-2 OS bone cells	[106]
poly-ε-caprolactone/chitosan nanofibres	Nanofiber mat as the sublayer, PU foam coated with ethanolic extract of propolis	Morphological and physico-mechanical properties Antibacterial activity (*Staphylococcus aureus, Escherichia coli*) Cytotoxicity assays (L929 Fibroblast) In vivo study	Bead-free, randomly oriented, continuous nanofibers, 207 nm; the layer composite porosity reduced to 68%, tensile strength to 6.21 MPa, elongation at break to 371%, contact angle to 58.6°; significant increase in swelling ratio; 17.7% degradation in 28 days ZOI 0.53 mm (*S. aureus*)/1.54 mm (*E coli*) Significantly enhances the cell viability Significantly and effectively accelerated the healing process	Antibacterial activity (ZOI: 0.53 mm for *S. aureus*, and 1.54 mm for *E. coli*); Significantly enhanced the viability of L292 fibroblasts; Significantly and effectively accelerated the healing process in in vivo murine models	[107]
Chitosan, 56–112 nm	Nanochitosan soaked in commercial PU foams	Morphological evaluation Phosphate removal Antimicrobial properties and coliform removal	Nanoparticles not agglomerated, no influence on morphological features Adsorption capacity ~ 17 mg/g Inhibition of coliform growth (>99%) Bacterial growth inhibition efficiency = 77.53%	Removed >99% of coliforms from the synthetic graywater Bacterial growth inhibition efficiency = 77.53%	[108]
MWCNTs	PU modified with amino acid (mixed in the polyol solution), soaked in dopamine solution, finally MWCNT solution added	Morphological evaluation Hydrophobicity and lipophilicity evaluation Oil sorption and oil water separation Antibacterial activity (*S. aureus, E. coli*)	MWCNT aggregates observed on surface, porous structure intact, water contact angle: 153°, lipophilic nature, over 97% efficiency in oil/water or organic solvent/water mixture separation, high antibacterial activity *E. coli* (>80%) and *S. aureus* (>75%); activity against Gram-negative bacteria was maintained at a high level after repeated use	High antibacterial activity against *E. coli* (>80%) and *S. aureus* (>75%); activity maintained at a high level against Gram-negative bacteria after repeated use	[109]

^1^ Abbreviations: NM—nanomaterial, NL—nanolignin, ZOI—zone of inhibition, NCl—nanoclay, MB—methylene blue, MO—methyl orange, SG—solvent green 3, MWCNTs—multi wall carbon nanotubes.

## Data Availability

Data are contained within the article.
